# One out of ten independent components shows flipped polarity with poorer data quality: EEG database study

**DOI:** 10.1002/hbm.26540

**Published:** 2023-12-09

**Authors:** Makoto Miyakoshi, Hyeonseok Kim, Masaki Nakanishi, Jason Palmer, Noriaki Kanayama

**Affiliations:** ^1^ Division of Child and Adolescent Psychiatry Cincinnati Children's Hospital Medical Center Cincinnati Ohio USA; ^2^ Department of Medicine University of Cincinnati College of Medicine Cincinnati Ohio USA; ^3^ Swartz Center for Computational Neuroscience Institute for Neural Computation, University of California San Diego La Jolla California USA; ^4^ School of Mathematical and Data Sciences West Virginia University Morgantown West Virginia USA; ^5^ Human Informatics and Interaction Research Institute, National Institute of Advanced Industrial Science and Technology (AIST) Tokyo Japan; ^6^ Center for Brain, Mind and KANSEI Sciences Research Hiroshima University Tokyo Japan

**Keywords:** EEG, ICA, indeterminacy, polarity, resting state, scalp topography

## Abstract

Independent component analysis (ICA) is widely used today for scalp‐recorded EEG analysis. One of the limitations of ICA‐based analysis is polarity indeterminacy. It is not easy to find detailed documentations that explains engineering solutions of how the polarity indeterminacy is addressed in a given implementation. We investigated how it is implemented in the case of EEGLAB and also the relation between the outcome of the polarity determination and classification of independent components (ICs) in terms of the estimated nature of the sources (brain, muscle, eye, etc.) using an open database of *n* = 212 EEG dataset of resting state recordings. We found that (1) about 91% of ICs showed positive‐dominant IC scalp topographies; (2) positive‐dominant ICs were more associated with brain‐originated signals; (3) positive‐dominant ICs showed more radial (peaked at 10–30 degrees deviations from the radial axis) dipolar projection pattern with less residual variance from fitting the equivalent current dipole. In conclusion, using the EEGLAB's default ICA algorithm, one out of 10 ICs results in flipping its polarity to negative, which is associated with non‐radial dipole orientation with higher residual variance. Thus, we determined EEGLAB biases toward positive polarity in decomposing high‐quality brain ICs.

## Graphical abstract

Polarities of independent components (ICs) are indeterminant. We found about 9% of ICs flip the initial tentative positive polarities to negative polarities when the algorithm converges. The polarity‐flipping ICs show less physiological validity. The IC polarity could be a novel criterion for evaluating ICs.

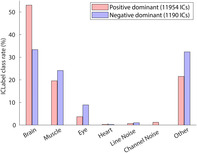

## 1 INTRODUCTION

The polarity of the scalp‐recorded EEG relates to the cytoarchitecture of the generative mechanism of EEG. In the field of computational neuroscience, these systems are modeled as follows (Neymotin et al., 2020): Pyramidal cells in cortical layer 2/3 (supra‐granular) and layer 5 (infra‐granular) are the main contributors of the extracellular electric fields. Lemniscal thalamic inputs to these neurons cause current flow up the dendrites toward supra‐granular layers, while non‐lemniscal or cortico‐cortical inputs to these neurons cause current flow down toward the infra‐granular layers. Thus, synaptic inputs to proximal regions cause source of the current, while those to distal regions cause sink of the current when seen from the cortical surface. When independent component analysis (ICA) (Bell & Sejnowski, 1995; Comon, 1994) is applied to scalp‐recorded EEG signals (Delorme et al., 2012; Makeig et al., 1996; Onton & Makeig, 2006), however, a known issue of indeterminacy of IC polarity occurs (Cong et al., 2008). The problem is that a result from a positive spatial weight times positive time series data (e.g., 1 × 1) cannot be distinguished from a negative spatial weight times negative time series data (e.g., −1 × −1). This polarity indeterminacy becomes a practical problem when averaging ICA‐decomposed ERPs across ICs because substantial amplitude reduction could happen if the ERP polarities are randomly determined. Since there is no mathematical solution to ultimately determine the “correct” IC polarities, how to determine the polarities is an engineering question in which analysts should choose the most reasonable solution for each application.

Recently, our group reported one such solution using covariance maximization across ICs in the framework of generalized eigenvalue problems (Nakanishi & Miyakoshi, 2023). This solution is available for aligning polarities of multiple ICs, which may be useful at the stage of the group‐level analysis to minimize amplitude cancellations across the clustered ICs. However, the suggested solution does not address the issue of how the polarity of a single IC is determined when the computation of ICA converges. The issue of single‐IC polarity is, again, indeterminant by nature and must be solved as an engineering problem. However the common EEG analysis tools available today, such as EEGLAB which has been promoting the use of ICA on EEG (Delorme & Makeig, 2004), provide solutions without clear documentation of how the IC polarities are determined when iterative learning process is done. Thanks to the open‐source policy of the EEGLAB, we investigated the original code. We found that when the algorithm starts the iterative learning process, all the IC polarities are set to be positive: the polarities of the IC scalp topographies, which are columns of the mixing matrix (in the EEGLAB variables, EEG.icawinv) rendered on scalp electrode locations, are positive dominant, that is, the peak of the scalp topography is positive. However, the validity of this assumption has not been tested.

In the current study, we investigated the relation between IC polarities calculated with initial all‐positive condition (EEGLAB's default behavior with no alternative) and IC qualities assessed by established metrics and methods, particularly class labels generated by ICLabel (Pion‐Tonachini et al., 2019). The main motivation of the study is to clarify the origin of the IC polarities and evaluate its influence in terms of physiological validity. Another agenda based on more personal observations is that high‐quality brain ICs almost always seem to show positive‐dominant scalp topographies. If this hypothetical conclusion is true, the mechanism must be explained. Most critically, it would be of great importance to know whether this tendency comes from artificial settings of ICA, or genuine physiology plays some role in the process. To answer to this question, we used an open‐source EEG database (Babayan et al., 2019) that provides over 210 datasets of 62‐channel scalp‐recorded EEGs to determine definitive observations to answer our questions.

## 2 MATERIALS AND METHODS

### 2.1 Subjects

We used the Leipzig Study for Mind–body‐Emotion Interaction dataset (Babayan et al., 2019). The exclusion criteria were as follows.

- Diagnosis of hypertension without intake of antihypertensive medication.
- Any other cardiovascular disease (current and/or previous heart attack or congenital heart defect).
- History of psychiatric diseases that required inpatient treatment for longer than 2 weeks, within the last 10 years (psychosis, attempted suicide, post‐traumatic stress disorder).
- History of neurological disorders (multiple sclerosis, stroke, epilepsy, brain tumor, meningoencephalitis, severe concussion).
- History of malignant diseases.
- Intake of one of the following medications (centrally active medication, beta‐ and alpha‐blocker, cortisol, any chemotherapeutic or psychopharmacological medication).
- Positive drug anamnesis (extensive alcohol, MDMA, amphetamines, cocaine, opiates, benzodiazepine, cannabis).
- MRI exclusion criteria (metallic implants, braces, nonremovable piercings, tattoos, pregnancy, claustrophobia, tinnitus, surgical operation in the last 3 months).
- Previous participation in any scientific study within the last 10 years.
- Previous or current enrollment in undergraduate, graduate, or postgraduate psychology studies.

After further excluding cases of recording failures due to technical problems, a total of 212 datasets were imported and preprocessed. The demographic information of the subjects included is as follows: 134 males; Age, *M* = 39.3 years (SD 20.3); Handedness, 188 right‐handed, 20 left‐handed, 4 ambidextrous. Note that the age information was provided for every 5 years tier, so the center of the bin was used for the representative value. For example, a participant in a tier of 20–25 years old was registered as 22.5 years old.

### 2.2 Ethics statement

The original data collection by Babayan and colleagues was carried out in accordance with the Declaration of Helsinki and the study protocol was approved by the ethics committee at the medical faculty of the University of Leipzig (reference number 154/13‐ff). In downloading the dataset, we confirmed that the data were de‐identified.

### 2.3 Task

Resting‐state tasks with eyes open and closed were used. The recording session was divided into two blocks: The first 8 min of eyes closed block followed by the second 8 min of eyes open block.

### 2.4 EEG recordings

Scalp EEG was recorded from the following 64 locations according to the international 10–10 system (Oostenveld & Praamstra, 2001): Fp1, Fp2, F7, F3, Fz, F4, F8, FC5, FC1, FC2, FC6, T7, C3, Cz, C4, T8, CP5, CP1, CP2, CP6, AFz, P7, P3, Pz, P4, P8, PO9, O1, Oz, O2, PO10, AF7, AF3, AF4, AF8, F5, F1, F2, F6, FT7, FC3, FC4, FT8, C5, C1, C2, C6, TP7, CP3, CPz, CP4, TP8, P5, P1, P2, P6, PO7, PO3, Poz, PO4, PO8, and FCz (the initial reference, which will be recovered at the cost of VEOG; see below). The online EEG data were recorded with a band‐pass filter between 0.015 Hz and 1 kHz with a 2500 Hz sampling rate and 0.1 μV resolution.

### 2.5 EEG preprocessing

EEG signals were downsampled to 250 Hz. The canonical electrode locations on the Montreal Neurological Institute head template were used (Collins et al., 1994; Evans et al., 1993). A high‐pass filter (FIR, Hamming, cut‐off frequency 1.5 Hz@‐6 dB, transition bandwidth 1 Hz) was applied. For the subsequent data cleaning stage, the EEG data were divided into the eyes open and closed data to be cleaned separately. EEGLAB plugin clean_rawdata() was applied with artifact subspace reconstruction with a cutoff threshold SD = 20 (Anders et al., 2020; Chang et al., 2018; Chang et al., 2020; Kothe & Jung, 2016; Kothe & Makeig, 2013). The separated data were combined again. The EEG data were re‐referenced to the average of the all the scalp electrodes plus the initial reference (i.e., continuous zeros) (Kim et al., 2023). In doing so, the initial reference electrode FCz was recovered while VEOG was discarded to keep the data ranked full. The adaptive mixture independent component analysis was applied (Palmer et al., 2016). At the first 15 iterations (max 2000), outlier data points larger than 3 SD were discarded for every iteration. EEGLAB plugin ICLabel (Pion‐Tonachini et al., 2019) was applied to probabilistically classify Ics into classes of brain, eye, muscle, heart, line noise, single channel noise, and others. The principle of how ICLabel works is as follows. First, over 200,000 ICs from more than 6,000 EEG sessions were collected to form a database. Then, these IC were manually labeled using an online crowd‐sourced solution. Finally, a weighted convolutional neural network learns the relation between the IC properties (IC scalp topography, power spectral density, and autocorrelation function) and human ratings to build a classifier that can generalize the learning results to a new input. Finally, equivalent current dipole models were fit to each IC scalp topographies (i.e., columns of ICA's mixing matrix rendered to scalp electrode locations) using Fieldtrip (Oostenveld et al., 2011) and bilateral symmetrical dipole fitter (Piazza et al., 2016).

### 2.6 EEG analysis

**FIGURE 1:**
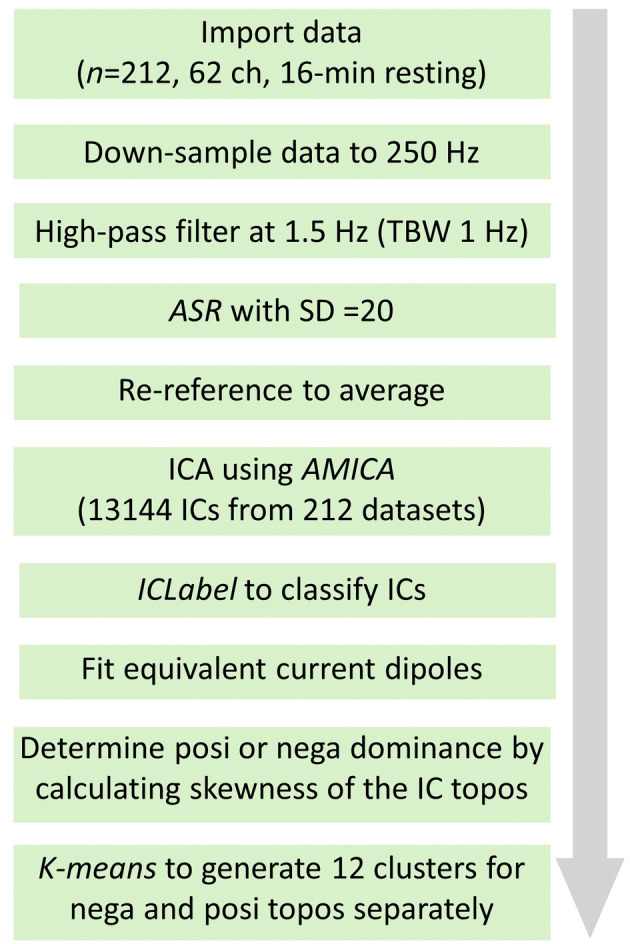
EEG preprocessing pipeline. ASR, artifact subspace reconstruction; AMICA, adaptive mixture independent component analysis; ICLabel, an EEGLAB plugin (Pion‐Tonachini et al., 2019).

Figure 1 shows a schematic illustration of the data preprocessing pipeline. To determine whether the obtained IC scalp topographies are positive‐ or negative‐dominated, skewness of the data distribution across scalp electrodes was calculated for each IC. The skewness of the IC scalp topographies was calculated using MATLAB function *skewness*(). Positive skewness indicates the obtained IC scalp topographies are positive‐dominant. The radiality of the fitted equivalent current dipoles was quantified as follows. The radial axes were defined by vectors originating from [0 0 0] of MNI's template brain's coordinate system to the location of the fitted dipoles. Angular differences between the dipole moment and radial axes were calculated to evaluate the radiality of the fitted dipoles. A smaller angular difference indicates closer to radial orientation. Radial dipole orientation indicates the estimated current source is localized on the surface of continua of neocortical gyral crowns (Nunez & Srinivasan, 2006), which verifies the physiological validity of the decomposed ICs.

### 2.7 Statistics

The k‐means algorithm (Forgy, 1965; Lloyd, 1982) was used to classify the IC scalp topographies into 12 clusters according to their similarities. The number of clusters 12 was determined to produce a convenient coarse‐grain view in a 3 × 4 grid plot. The clustering was done separately for ICs with positive and negative dominance in IC topographies for comparison. No inferential statistics were used to draw conclusions. This was why we chose the EEG database with a relatively large number of datasets (*n* = 212) to obtain robust observations.

## 3 RESULTS

**FIGURE 2:**
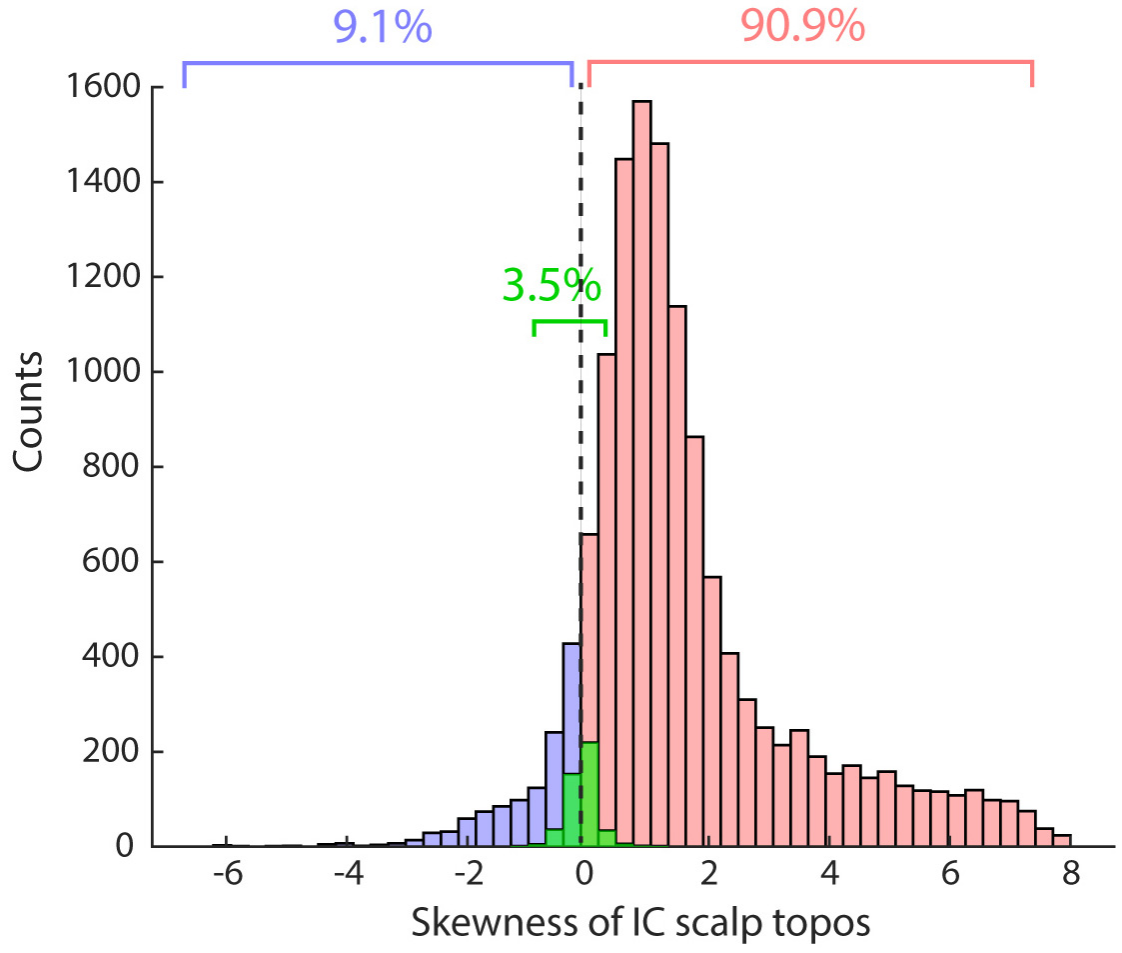
The distribution of the skewness of IC scalp topographies. There were a total of 13,144 ICs (62 ICs × 212 subjects). In the figure, 90.9% of the ICs (red) showed positive dominance and positive skewness, while 9.1% of the ICs (blue) showed negative dominance and negative skewness. About 3.5% of the ICs (green) showed a mismatch between the signs of the skewness and dominance of IC scalp topography.

A total of 13,144 ICs (62 ICs × 212 subjects) and corresponding IC scalp topographies were generated. The distribution of the skewness is shown in Figure 2. The descriptive statistics revealed that 90.9% of the ICs showed positive dominance and positive skewness, while 9.1% of the ICs showed negative dominance and negative skewness. About 3.5% of the ICs showed a mismatch between the signs of the skewness and dominance of IC scalp topography, which confirms that the strategy to use skewness as a metric to determine the dominant polarity was mostly successful. It became clear that more than 90% of ICs have positive dominance in their scalp topographies. This was expected as the initial conditions for these polarities are hard‐coded to be positive. Thus, only 9.1% of ICs flipped their polarities as a result of the full ICA process.

**FIGURE 3:**
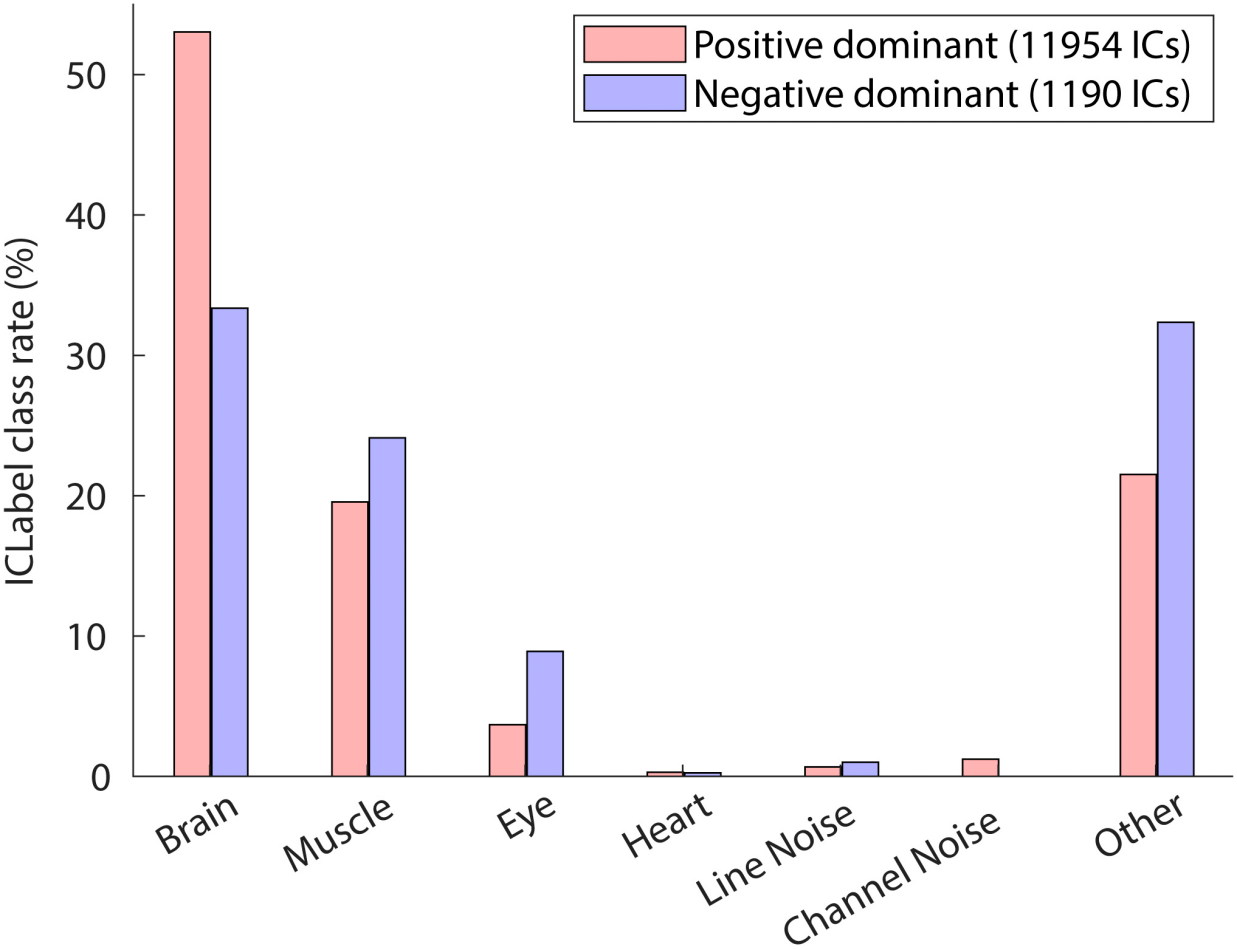
Comparing ICLabel's class rates between IC groups with positive and negative dominance on the IC scalp topographies.

In the next step, we compared the rate of the IC classes determined by the ICLabel algorithm (Pion‐Tonachini et al., 2019). The results are shown in Figure 3. More than 50% of the positive‐dominant ICs were classified as ‘Brain’, while less than 35% of the negative‐dominant ICs were classified as ‘Brain’. In contrast, negative‐dominant ICs showed generally higher rates for non‐brain classes than positive‐dominant ICs. The result indicates that negative‐dominant ICs are more frequently associated with poor quality in brain signal decomposition.

**FIGURE 4:**
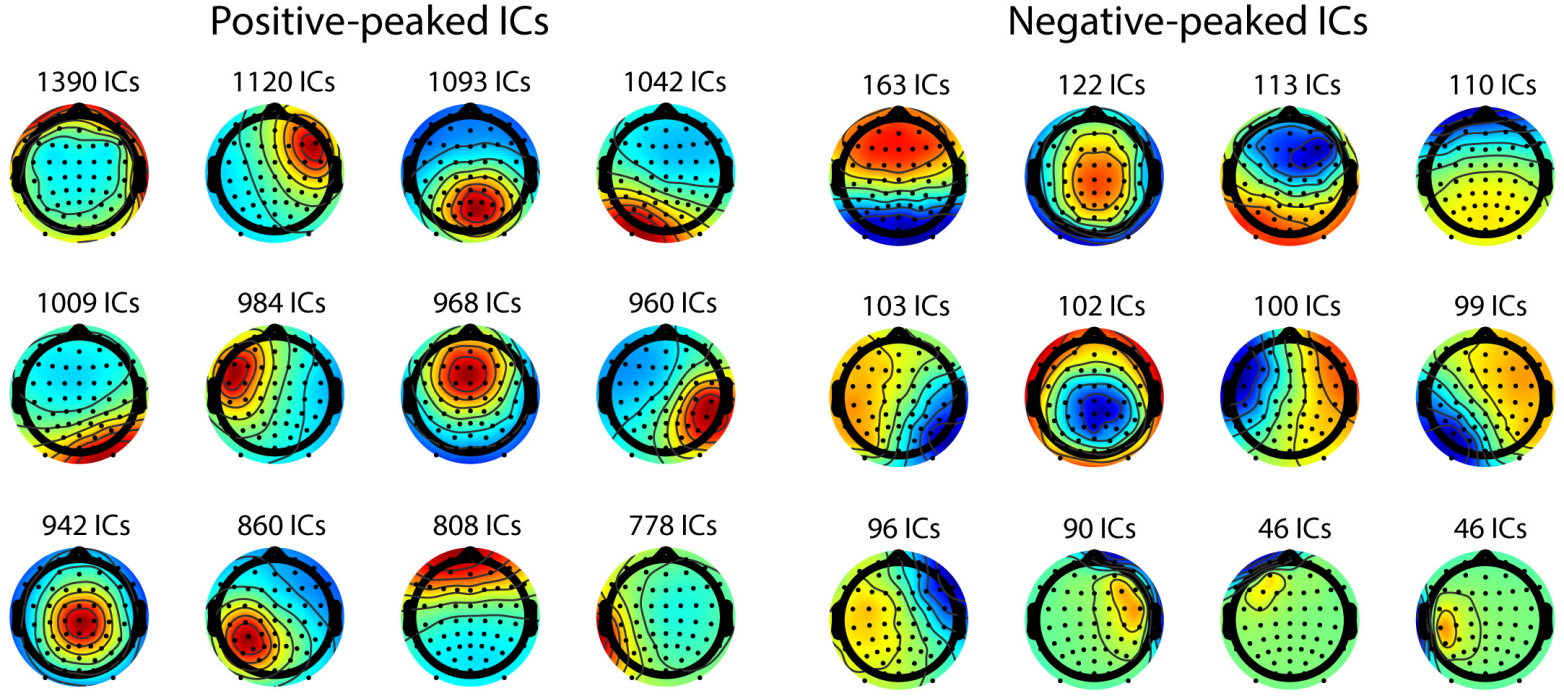
k‐means clustering applied to positive‐ and negative‐dominant ICs separately. Note that the positive‐dominant ICs show more radial dipole projection patterns, while the negative‐dominant ICs show more tangential projection patterns.

To visually confirm the differences in the scalp topographies between the positive‐ and negative‐dominant ICs, the obtained IC scalp topographies were clustered into 12 clusters using k‐means. Figure 4 shows the results. The noticeable difference in this visual comparison is the *angle* of the dipoles: the positive‐dominant ICs seem to have radial dipoles. In contrast, the negative‐dominant ICs seem to have tangential dipoles.

**FIGURE 5:**
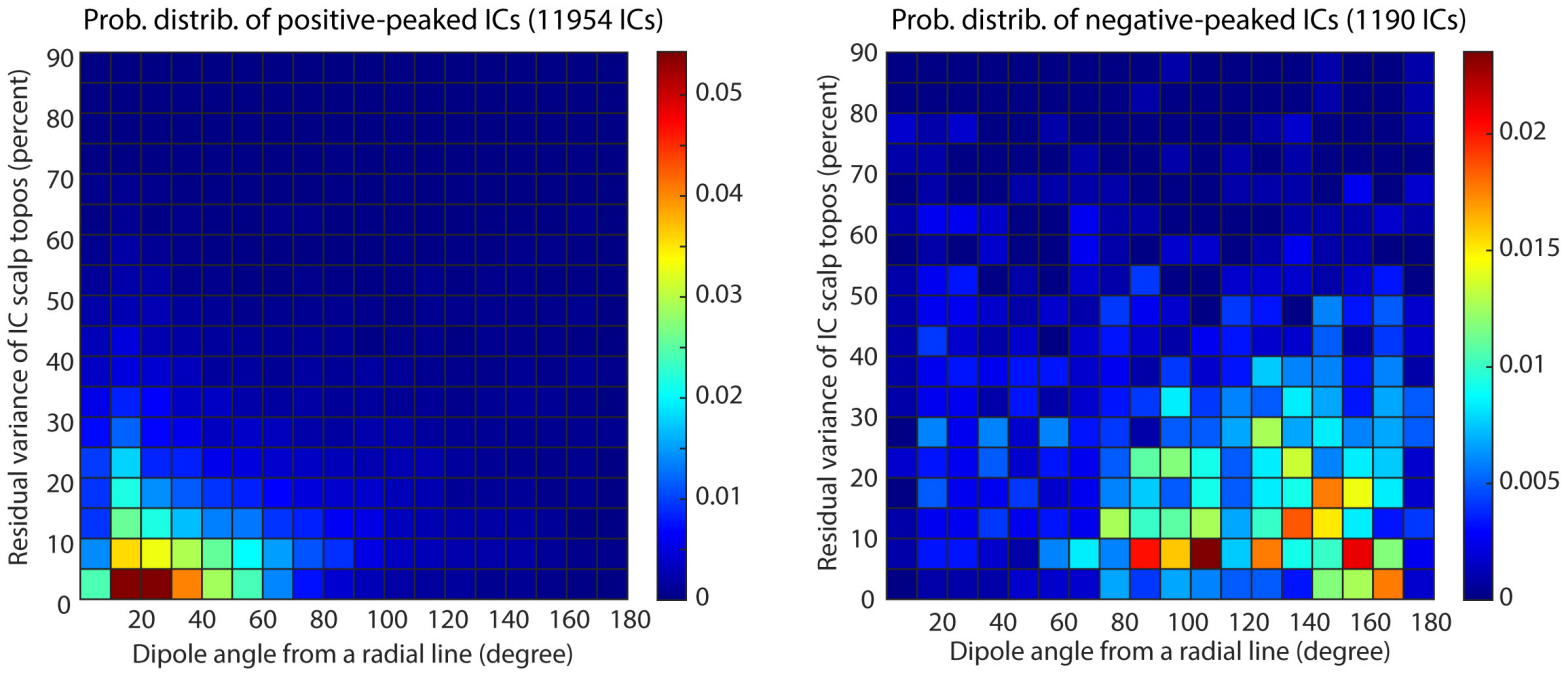
The residual variance from fitting equivalent current dipole to IC scalp topography plotted as a function of the dipole angle deviated from radial axes. The left and right plots compare the results for positive‐ (left) and negative‐ (right) dominant ICs. The normalization is applied so that all grid values sum to 1 for each plot.

To quantify the visual impression of the dipole angle difference between the positive‐ and negative‐dominant ICs, we designed a visualization of probability density on the plane defined by dipole angle defined as a deviation from a radial line and residual variance of the IC scalp topographies compared with theoretical projection from the estimated dipole. The results are shown in Figure 5. As demonstrated in our previous study (Delorme et al., 2012), the residual variance is the measure of the *dipolarity* of ICs which has been interpreted as physiological validity. The probability density distribution of the positive‐dominant ICs shown in Figure 5 left showed a clear unimodal pattern: the peak probability resides below 5% of the residual variance and within the 10–30 degrees deviation range from the radial projection axis. In contrast, the probability density distribution of the negative‐dominant ICs showed broad distribution between the ranges of up to 30% of residual variance and between 80 and 170 degrees of deviation from the radially projecting axis. Note also that the area in which positive‐dominant ICs showed peak probability density is quiet in the corresponding area in the negative‐dominant ICs.

## 4 DISCUSSION

Investigating the IC polarity patterns using the relatively large empirical EEG dataset yielded the following observations: (1) About 91% of ICs showed positive‐dominant IC scalp topographies when initial polarity is set to positive for all ICs; (2) Positive‐dominant ICs are more associated with brain‐originated signals; (3) Positive‐dominant ICs showed more radial (peaked at 10–30 degrees deviations) dipolar projection pattern with less residual variance from fitting the equivalent current dipole. These results support the general view that negative‐dominant ICs are minorities with poor signal quality in brain signal decomposition.

The final results showed most ICs had the initial polarity values. If we set the initial polarities to all negative, we would see 91% of ICs with negative‐dominant topographies with polarity‐inverted IC activation time series data. As the initial polarities determine the final polarities for most ICs, it is meaningless to argue the absolute polarity. Instead, the critical finding is that while the majority of the ICs (91% in the current study) remain the same polarities as the initial values, the remaining ICs do flip the polarities during the process of decomposition, and the polarity flippers are associated with poor signal/decomposition quality. As far as we know, this property of ICA has never been documented. This observation adds a new criterion to evaluate ICs: high‐quality signals/decompositions show polarities consistent with the initial values. In the case of implementation in EEGLAB, the initial values are positive‐dominant, hence high‐quality signals/decompositions are more likely to show radial and dipolar projections with positive dominance.

The result also confirms that the ICA‐based EEG decomposition primarily captures gyral sources. Given the fact that the total area of gyral crowns is about one‐third of that of the entire cortical surface (Standring, 2020), if ICA were equally sensitive to sulcal sources, the result in Figure 5 would have shown another peak, with even higher value, at around 90°. Our result justifies the view that sensitivity to sulcal sources may not be very important in analyzing human EEG because of (1) cancellation of the electric fields between the two cortices facing each other and (2) larger distance from the scalp (Nunez & Srinivasan, 2006). The peak density at around 90° with relatively low residual variance in the negative‐dominant ICs seems to point to genuine sulcal EEG sources whose scalp topographies should show both positive and negative peaks.

The selection of the reference electrode affects EEG polarity in scalp recording case. This reference potential problem may be relatively reasonably addressed by using either an average reference for high‐density EEG systems or the REST algorithm (Yao, 2001) for re‐referencing (Nunez, 2010). Though ICA results are invariant to the choice of reference electrodes after subtracting mean values topography‐wise, to verify IC polarities, it seems required at this point to verify the polarities against known examples if they are available. For example, suppose an IC is identified as a significant contributor to classical P300 in terms of its latency and scalp distribution. In that case, the polarity should be set so that the waveform of the IC ERP also shows P300, not N300 (Nakanishi & Miyakoshi, 2023). This empirical workaround may be used as long as well‐established examples are available. In the case of continuous data decomposition, such as resting state, this approach does not work. Although IC polarities do not seem to matter very frequently for continual data analyses, it is a problem for which we do not have a solution, and we do not have a reasonable way to justify our default choice, such as starting from all‐positive‐dominant scalp topographies.

It may be worth mentioning that ICA results are invariant to the choice of reference because re‐referencing and ICA are both linear operations. One exception is that mean values across all the electrodes in an IC scalp map can vary depending on the choice of reference. The average reference method forces every IC scalp topographies to approach close to zero mean. Technically, the deviation is controlled to be 1/(number of channels +1) of the mean value of each IC topography (Kim et al., 2023). For other choices of reference electrodes, scalp topographies could be dominated by general positivity or negativity that appears as “all red” or “all blue” using the conventional color scheme, respectively. Using average reference is one of the reasonable solutions to produce IC scalp topographies that are well‐balanced between positivity and negativity.

We speculate why poor decompositions tend to have negative‐dominant scalp topographies as follows. Such poor decompositions do not have unimodal (or bimodal for the case of major tangential sources) scalp topographies; in other words, “residual variances” from fitting the radial (or tangential) equivalent current dipole become high (Delorme et al., 2012), which, in turn, leads to IC scalp topographies have multiple positive and negative local peaks. In this case, nonpositive dominance can be understood as an indicator of poor component quality. Usually, ICs with high variance accounting are more likely to reflect brain signals because in EEG signals high amplitude generally means high SNR (Nunez & Srinivasan, 2006). ICs with low variance accounting usually suffer from poor component quality and they are always there. However, they account for progressively smaller data variance, which may be understood as residuals from decomposing main signals. Perhaps ICA uses those low‐variance residuals to make ICA work as a complete linear decomposition; we can imagine ICA uses them to cancel out residuals to “make ends meet” in the process of linear decomposition. If our speculation is correct, using the relative dominance of ICs, either in amplitude or valiance, as an additional evaluation criterion for physiological validity seems possible. This viewpoint appears missing from conventional studies using ICA. Our study provides partial evidence that ICs with low variance are less reliable in terms of physiological validity and not purely procedural reproducibility, which may be used for future studies to test the validity of ICA and ICASSO (Artoni et al., 2014; Groppe et al., 2009; Himberg et al., 2004; Himberg & Hyvarinen, 2003; Hyvärinen et al., 2001). In the conventional ICA applications, there was no explicit consensus that ICs with trivial variance explained also have trivial physiological validity or significance (Delorme et al., 2012; Onton & Makeig, 2006). However, the current study demonstrated that ICs with low variance do not have the same level of physiological validity at least in terms of the dipole angle analysis. Because ICA can be also understood as a mode decomposition technique (Friston, 1998), investigating how the quality of decomposition relates to the variance of components in future studies seems to produce valuable insights.

In conclusion, we clarified that EEGLAB's default ICA sets all the IC polarities to be positive, leading one of ten ICs to flip its polarity to negative. We found that negative‐dominant ICs are associated with poorer data quality. The positive‐dominant ICs show highly radial projection patterns with low residual variance from fitting equivalent current dipoles. This pattern does not fit the negative‐dominant ICs. Thus we determined EEGLAB biases toward positive polarity in decomposing high‐quality brain ICs.

## CONFLICT OF INTEREST STATEMENT

The authors declare no conflicts of interest.

## Data Availability

The data that support the findings of this study are available in MPILMBB LEMON Data at https://www.gwdg.de/. These data were derived from the following resources available in the public domain: Babayan et al. (2019), https://www.nature.com/articles/sdata2018308
